# Quantitative genetics in the monk parakeet (*Myiopsitta monachus*) from central Argentina: Estimation of heritability and maternal effects on external morphological traits

**DOI:** 10.1371/journal.pone.0201823

**Published:** 2018-08-03

**Authors:** Juan J. Martínez, María C. de Aranzamendi, Enrique H. Bucher

**Affiliations:** 1 Instituto de Ecorregiones Andinas, Universidad Nacional de Jujuy, CONICET. C, San Salvador de Jujuy, Argentina; 2 Instituto de Diversidad y Ecología Animal (CONICET-UNC) and Facultad de Ciencias Exactas, Físicas y Naturales, Universidad Nacional de Córdoba, Córdoba, Argentina; 3 Centro de Zoología Aplicada, Instituto de Diversidad y Ecología Animal (CONICET-UNC) and Facultad de Ciencias Exactas, Físicas y Naturales, Universidad Nacional de Córdoba, Córdoba, Argentina; Fred Hutchinson Cancer Research Center, UNITED STATES

## Abstract

The monk parakeet (*Myiopsitta monachus*) is a South American species unique among parrots; it builds communal stick nests that allow independence from tree or cliff cavities required by most parrots. As a very successful invasive species, it has expanded into several countries around the world. Questions remain around the factors that allowed this species to be such a successful invader in its native range as in other countries, and particularly the extent that evolutionary processes may be involved in adapting to new areas. Along with this line of analysis, we focused on assessing whether morphological characteristics are sufficiently heritable, and therefore responsive to selection. As the first step in this direction, we have estimated heritability of monk parakeet in six external morphological traits considered of potential adaptability value. Samples were obtained in the province of Córdoba in central Argentina. Data from seven microsatellites were used to determine the familial relationships among individuals. Heritabilities and genetic correlations were estimated by means of animal models. We found evidence for significant heritability in the six traits measured, particularly in weight, tarsus length and bill width. We also found evidence of maternal effects on morphological traits, particularly in the traits with lower heritability: wing length, bill length and tail length. Genetic correlations between traits were significant and associated with phenotypic correlations, suggesting that these traits are constrained in terms of evolutionary potential, whereas the amount of additive genetic variance in weight, tarsus length and bill width indicate that these traits could be responsive to selection.

## Introduction

The monk parakeet (*Myiopsitta monachus*) is a South American species unique among parrots since it builds communal stick nests. Monk parakeets breed and roost in large, fully enclosed communal nest that allow independence from tree or cliff cavities, the required breeding habitats by most parrots. Each pair builds its brooding chamber where they lay the eggs. Nests typically include 1–4 chambers [1, 2].

This nesting habitat flexibility may contribute substantially to the considerable success of the monk parakeet as an invasive species. It already expanded into several countries in South, Central and North America, as well as in Europe, the Caribbean and Japan [3]. The monk parakeet is also expanding in its native distribution areas [4].

The monk parakeet creates human-wildlife conflict as it causes crop damage and also builds large nests in electricity lines increasing their operation and maintenance costs [5]. Accordingly, the monk parakeet has attracted considerable attention and research effort, not only because of practical management needs, but also due to its unique ecological and behavioral characteristics [1]. In this sense, identification of the species’ characteristics related to its success as an invasive species is of particular interest.

It has been suggested that the ecological success of monk parakeets appears related to behavioral flexibility and dietary opportunism favored by high intelligence and morphological adaptations, mainly in bill and foot structure [4]. In fact, most parrot species use bills and feet structures to obtain food, nest building, and even to take different tools [6–8].

Other morphological traits that deserve ecological and evolutionary consideration include weight, wing length, tarsus length, and tail length. Bill traits are associated with many characters, including foraging and song performance in birds. Weight is a general size measurement, a more condition-dependent trait. Wing and tail length are connected to flight performance. Tarsus length is a good proxy for overall structural size in birds, because it is a skeletal measurement [9].

Concerning the monk parakeet, an open research question relates to the extent that these morphological characteristics are sufficiently heritable, and therefore responsive to selection under the variable environmental conditions found by this species in both native and the invasive range. In this respect, consideration should be given to the fact that rapid morphological changes could be due to phenotypic plasticity and/or selection [10]. If traits have been under strong directional selection, it tends to eliminate genetic variation in phenotypic traits in direct proportion to their effect on fitness [11–13]. Alternatively, low heritability could be the result of increased residual variance, rather than reduced genetic variance [14–16].

Additional factors may obscure the estimation of heritability values. One of these is the occurrence of extra-pair paternity (EPP), which causes a misidentification of paternity and can bias the estimation of heritability [17]. The non-genetic resemblance between parents and offspring could also lead to incorrect heritability estimation values. For example, the phenotypic dissimilarity between the cuckolded male and the genetic father might result in an underestimation of the heritability value [18]. Gonçalves da Silva et al. [19] did not find cases of EPP in any of the three studied populations of monk parakeets (one in the species’ native area in Argentina and two in invasive areas in the USA), but Martínez et al. [20] found evidence of EPP (40% of breeding chambers) and intra-brood parasitism (3% of chambers) in a population from central Argentina.

Moreover, another non-genetic cause of resemblance between parents and offspring is the existence of maternal effects, when the phenotype of an individual is determined not only by its own genotype and the environmental conditions it experience during development but also by the phenotype or environment of its mother [12]. This factor could be significant in the majority of bird species since they care for their young for an extended period [21].

In summary, assessing the role played by morphological characteristics in the ecological success of the monk parakeet requires confirming their heritability as a first step. In the present study we present an estimation of the heritability of six morphological traits (weight, wing length, tarsus length, tail length, and bill length and width) in a monk parakeet population from central Argentina. We used animal models to estimate quantitative genetic parameters from a reconstructed genetic pedigree of individuals by genotyping seven microsatellite loci, thus avoiding the influence of EPP in heritability estimation. Maternal and nest effects were also considered to account for non-genetic resemblance between individuals.

## Material and methods

This study was carried out in strict accordance with the Guidelines for Ethical Research on Laboratory and Farm Animals and Wildlife Species and with the prior approval of the ethics committee of the Consejo Nacional de Investigaciones Científicas y Técnicas (CONICET) (Resolution No. 1047). The necessary permits were acquired from the Ministry of Environment of the Province of Córdoba, Argentina.

### Sampling sites and measurement of morphological traits

Fieldwork was carried out in central Argentina (Córdoba province), in an ecoregion originally characterized by a dry woodland forest (the Espinal ecoregion corresponding to the NT0801 world ecoregion according to Olson et al. [22]). The original vegetation has been cleared for agriculture to a large extent. Samples were collected in two localities from central Argentina (Córdoba province), 20 km apart: Marull (31° 40’ S, 62° 49’ W) and Miramar (32° 55’ S, 62° 40’ W). We consider that samples belong to a single population taking into account the short distance between sites and also that previous work found evidence of a lack of genetic structure and of homogeneity in allelic frequencies between these two sites [20].

Nests were examined during the last week of November and the first week of December, 2000. Nests were located in eucalyptus tree rows along fences, over 15 m above ground level. We reached the nests during the night using a cherry picker truck. Parakeets were trapped from the nests at night using a specially designed funnel net placed below each nest entrance [23]. During night time parakeets do not leave the nest even if under moderate disturbance, whereas during day time they disperse well before observers can even approach the nest.

Nestlings were removed by hand from the nest after capturing the adults. All individuals were kept in specially designed boxes during the remaining of the night to avoid stress and predation if released. Early in the following morning nestlings were relocated to their nest chambers, and the remaining individuals (able to fly) were released in the proximity of their nests.

We were unable to capture all adult individuals in nests, because some escaped when we were approaching the nests. However, we are certain that all of the trapped individuals were roosting in the chambers where they were captured as we did not find openings between chambers that could allow adults to move at the moment of trapping. No information is available on the social status of the trapped adults besides their chamber location.

A total of 28 nests were sampled (Miramar = 19, Marull = 9): 21 nests had a single chamber, six nests had two chambers, and one nest had four chambers. A total of 195 individuals were genotyped (154 nestlings, and 41 adults, including 21 candidate fathers, 20 candidate mothers). The following six measurements were taken from each individual: weight (in g), wing length (from the bend of the wing to the tip of the longest primary feathers), tarsus length (from the inner bend of the tibiotarsal articulation to the base of the toes), tail length (from the base of the tail to the tip of the longest feathers), and bill length and width (all in mm). Measurements were taken by the same person using calipers and rulers.

For testing heritability of morphological traits, we took into account the average mass at fledging in this species estimated to be between 88.7 and 105.5 g [24]. Accordingly, we decided to include all individuals whose weight was over 82 g, considering that this value corresponds to the minimum weight found in an adult in our sample, which is close to the cut-off value of 88 g estimated by Navarro and Bucher [24].

Pearson correlation test, in the Hmisc package in R [25, 26], was used to estimate the relationships between the traits in nestling data set. Adult sexual differences in the traits were evaluated using ANOVA and MANOVA for univariate and multivariate analyses, respectively. Assortative mating was analyzed by means of Pearson correlation test between trait values from males and females.

Analyses of animal models included 133 individuals with genetic pedigree information (122 both full-siblings and half sibling nestlings and 11 adults) and 86 founders (i.e., the individuals for which both the father and the mother are unknown) (see S1 Table for pedigree information); the remaining individuals sampled were not considered. Thus, the main contribution to heritability estimates appears to be based on between-nestlings relationships instead of parent-offspring relationships.

### DNA extraction, genetic analyses and pedigree estimation

As described in detail previously [20], we assigned relationship among individuals using seven microsatellite loci. Briefly, genomic DNA was extracted from blood samples and analyzed as in Martínez et al. [20]. Seven microsatellite loci were used for relatedness and parentage analyses: AgGT19, AgGT29, AgGT90 [27], MmGT046, MmGT054, MmGT057 and MmGT060 [28]. The combined exclusion probability with one-parent known was 0.985, while the combined probability of excluding two putative parents was 0.999. The sex of adults was determined molecularly as indicated in Griffiths et al. [29] using the specific markers P2 and P8 for ZW sexual chromosomes.

We estimated genetic relationships between individuals to reconstruct more accurately the population genetic pedigree (see S1 Table for pedigree information). Values of genetic relatedness among individuals were taken from Martínez et al. [20]. Presence of null alleles was investigated in the whole data set using the software Micro-Checker v2.2.3 [30]. The ML-Relate program [31] was used to adjust allelic frequencies for null alleles. Allelic richness, observed and expected heterozygosity, and tests for deviation from Hardy-Weinberg equilibrium and linkage disequilibrium were calculated with Arlequin 3.5 [32].

Full-sibs, and paternity and maternity of nestlings were identified by using the maximum likelihood method implemented in Colony 2.0.1.1 [33]. We used the full likelihood method option, the long option for the length of run and the allelic frequencies adjusted for null alleles. Longer runs (10 replicates) are more likely to find the maximum likelihood configuration. Finally, the possibility of parentage and sibship was excluded from the alternative locality. The probability threshold to assign parentage was 0.99. For further details, see Martínez et al. [20] as we estimated quantitative genetic parameters in the same individuals from that study.

### Heritability, maternal effect and genetic correlations

The narrow-sense heritability (*h*^*2*^) of a trait is the proportion of its total phenotypic variance that is determined by additive genetic variance, and that is available for selection to act upon [12]. Contributions of genetic and environmental effects on morphological traits were estimated using animal model analyses as implemented in MCMCglmm package in R [26, 34]. For univariate models, the posterior distribution was sampled every 100 iterations with a burn-in 100,000 for a total of 9,000 samples. For both *G* (random effects) and *R* (residuals) priors, we specified *V* = (trait’s variance*0.05) and *nu* = 1. The bivariate models were performed in nestling data set and sampled every 1,000 iterations with a burn-in of 400,000 for a total of 3,600 samples. For bivariate models, we used *V* = diag(2) and *nu* = 1.002 for *G* and *R* priors.

Variance parameters were estimated as the posterior mode with 95% credible intervals (CI) based on the posterior distribution of the parameter. At each MCMC iteration, all variance ratios and correlations were estimated based on variance-covariance components, thus providing a posterior distribution. The posterior distribution of heritability (*h*^*2*^) is equal to the ratio of *V*_*A*_ (additive genetic variance) to *V*_*P*_ (phenotypic variance). Additionally, using a bivariate form of the model, we calculated the genetic correlation (*r*_*G*_) between each pair of morphological traits from their genetic variance (*V*_*x*_ or *V*_*y*_) and covariance (Cov_xy_): *r*_*G*_ = *Cov*_*xy*_ / (*V*_*x*_ *V*_*y*_)^1/2^.

As variance parameters are bounded above zero, we estimated the importance of random effects by looking at the deviance information criteria (DIC) [35]. The DIC is analogous to the Bayesian version of Akaike information criterion (AIC). We used a delta DIC value under seven [36] to identify potentially important random effects.

## Results

### Phenotypic correlation and assortative mating

Summary statistics for the six morphological traits taken on our sample (parents and nestlings) are shown in Table 1. We found high levels of trait correlation between tail length and wing length (r = 0.71) and between wing length and bill length (r = 0.69). Length and width of the bill were moderately correlated (r = 0.32). Also, moderate correlations were found between tail length and bill length (r = 0.32), between bill width and wing length (r = 0.33) and bill width and tail length (r = 0.27; Table 2). Low but still significant phenotypic correlation was found between weight and wing length (r = 0.23) and between weight and bill width (r = 0.20).

**Table 1:** Summary statistics of monk parakeet’s morphological traits in adults and nestlings used in animal models. Weight in g and the rest of the traits in mm. SD = standard deviation.

			Adults					Nestlings		
Trait	N	Mean	SD	Min	Max	N	Mean	SD	Min	Max
Weight	46	96.02	6.35	82	115	121	101.35	8.24	82	121
Wing length	46	145.5	5.93	128	156	122	95.88	33.67	34	192
Tarsus length	46	19.18	1.46	17	25	122	18.44	1.35	14	21.9
Bill length	46	12.65	1.99	9	18	122	9.82	2.64	6	16.7
Bill width	46	11.76	0.62	11	13.2	122	11.31	0.52	9.1	12.2
Tail length	46	145.22	12.63	102	167	122	64.52	25.56	11	146

**Table 2:** Phenotypic correlations (Pearson) between six external morphological traits measured in nestlings of monk parakeet. Pearson’s R is shown below the diagonal. Significant values are shown in bold.

**Traits**	**Weight**	**Wing length**	**Tarsus length**	**Bill length**	**Bill width**	**Tail length**
**Weight**		*	ns	ns	*	ns
**Wing length**	**0.23**		ns	***	***	***
**Tarsus length**	0.11	0.07		ns	ns	ns
**Bill length**	0.12	**0.69**	0.06		***	***
**Bill width**	**0.20**	**0.33**	0.17	**0.32**		***
**Tail length**	0.15	**0.71**	-0.002	**0.50**	**0.27**	

ns: not significant;

* p < 0.05;

** p < 0.01;

*** p < 0.001

There was significant assortative mating in two of six traits: weight and bill length have high and significant positive correlation in both parents (N = 6 pairs) (r = 0.933; p = 0.0065 and r = 0.887; p = 0.018, respectively). The ANOVA for univariate traits (p ranged from 0.055 to 0.728) and MANOVA (Wilk’s lambda = 0.745; df1 = 6; df2 = 19; p = 0.407) indicated no sexual differences in the values of the different traits measured in adults.

### Univariate decomposition of variance

**Fig 1:**
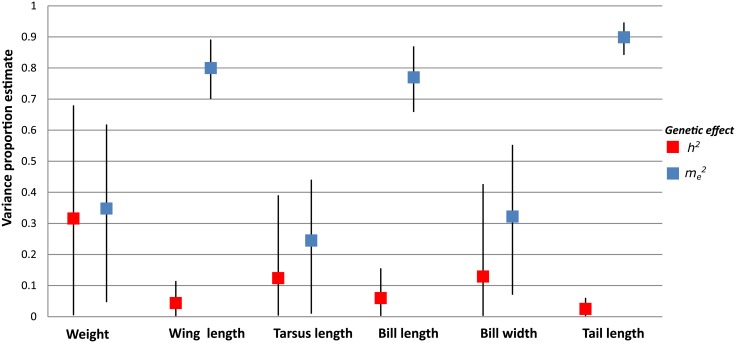
Narrow-sense heritability (*h*^*2*^) and maternal effect (*me^2^*) of six morphological traits in the monk parakeet based on animal models.

In the six morphological traits, all animal models that included additive genetic and maternal effects fell within a delta DIC of seven (Table 3) being equally plausible as the best model. We found nonzero heritability in all the morphological traits (from 0.023 to 0.314; Fig 1, S2 Table). The heritability of weight equaled *h*^*2*^ = 0.314 (95% CI = 0.005–0.679), for the tarsus length the heritability equaled *h*^*2*^ = 0.122 (95% CI = 0.004–0.390) and for bill width the heritability equaled *h*^*2*^ = 0.127 (95% CI = 0.003–0.426). Maternal effects were particularly high in the following traits: wing length (*m*_*e*_^*2*^ = 0.800, 95% CI = 0.701–0.891), bill length (*m*_*e*_^*2*^ = 0.77, 95% CI = 0.659–0.869) and tail length (*m*_*e*_^*2*^ = 0.899, 95% CI = 0.843–0.946; Fig 1, S2 Table).

**Table 3:** Model selection of univariate estimations of genetic variance. Model selection based on deviance information criterion (DIC). Most parsimonious models are highlighted in bold.

Trait	Model (random effects)	DIC	Δ DIC
*Weight*	Additive genetic	**833.25**	–
	Additive genetic + maternal	**838.42**	**5.17**
	Additive genetic + nest	845.65	12.4
	Additive genetic + nest + maternal	845.66	12.41
*Wing length*	Additive genetic + maternal	**1137.81**	–
	Additive genetic + nest + maternal	**1138.82**	**1.01**
	Additive genetic + nest	1244.65	106.84
	Additive genetic	1262.03	124.22
*Tarsus length*	Additive genetic + maternal	**431.98**	–
	Additive genetic + nest + maternal	**433.96**	**1.98**
	Additive genetic	**434.61**	**2.63**
	Additive genetic + nest	440.16	8.18
*Bill length*	Additive genetic + maternal	**459.09**	–
	Additive genetic + nest + maternal	**460.19**	**1.1**
	Additive genetic + nest	566.62	107.53
	Additive genetic	594.69	135.6
*Bill width*	Additive genetic + maternal	**158.69**	–
	Additive genetic + nest + maternal	**160.33**	**1.64**
	Additive genetic	**163.81**	**5.12**
	Additive genetic + nest	170.51	11.82
*Tail length*	Additive genetic + maternal	**1090.56**	–
	Additive genetic + nest + maternal	**1090.69**	**0.13**
	Additive genetic + nest	1288.81	198.25
	Additive genetic	1306.09	215.53

### Genetic correlations among morphological traits

Bivariate animal models of nestlings that included only additive genetic variance were by far the best models according to DIC values, although some exceptions occurred (Table 4). We found significant genetic correlations between wing length and bill length, wing length and bill width and wing length and tail length. Also, genetic associations were positive between bill length and bill width, bill length and tail length and bill width and tail length. We found only one significant nest correlation between wing length and tail length (Table 4).

**Table 4:** Genetic, maternal and nest correlation between each pair of morphological traits measured in nestlings. All correlations are given with 95% credible intervals. Significant correlations are highlighted in bold.

*Traits*	*Genetic correlation*	*Maternal correlation*	*Nest correlation*
Weight/Wing length	0.179 (-0.022 to 0.363)	–	–
Weight/Tarsus length	0.153 (-0.164 to 0.467)	–	–
Weight/Bill length	0.135 (-0.064 to 0.325)	–	–
Weight/Bill width	0.285 (-0.069 to 0.635)	0.089 (-0.524 to 0.662)	–
Weight/Tail length	0.033 (-0.242 to 0.308)	–	0.658 (-0.534 to 0.998)
Wing length/Tarsus length	-0.082 (-0.710 to 0.683)	-0.043 (-0.827 to 0.771)	0.195 (-0.488 to 0.880)
Wing length/Bill length	**0.569 (0.416 to 0.707)**	–	–
Wing length/Bill width	**0.346 (0.078 to 0.614)**	–	0.174 (-0.323 to 0.682)
Wing length/Tail length	**0.644 (0.494 to 0.791)**	–	**0.912 (0.733 to 0.999)**
Tarsus length/Bill length	0.064 (-0.208 to 0.321)	–	–
Tarsus length/Bill width	0.260 (-0.113 to 0.599)	–	–
Tarsus length/Tail length	0.033 (-0.228 to 0.277)	–	–
Bill length/Bill width	**0.263 (0.024 to 0.482)**	–	–
Bill length/Tail length	**0.478 (0.334 to 0.621)**	–	–
Bill width/Tail length	**0.398 (0.129 to 0.642)**	–	0.049 (-0.512 to 0.604)

## Discussion

Morphological traits that cause variation in fitness have the potential to evolve over time if those traits are sufficiently heritable and responsive to selection [37]. Our results provide evidence for significant heritability in the six traits measured (weight, wing length, tarsus length, tail length, and bill length and width) and therefore open the possibility of these traits being under natural selection pressure, particularly during adaptation to new environments occupied by this species. They also show that a higher proportion of the phenotypic variance of weight, tarsus length and bill width is determined by additive genetic variance, rather than by the influence of the environmental conditions to which each individual is exposed (e.g., residual variance). The heritability of the studied traits indicate the possibility of being shaped by natural selection in response to changing environmental conditions (including global climate change) in both the native and invasive species range. This could be supported by the findings of Le Gros et al. [10], who found a rapid morphological divergence (< 50 years) in external traits and in bill shape of *Psittacula krameri* in invasive populations with respect to native ones.

### Nest and maternal effects

Common environmental effects can have an important contribution to the estimation of heritability, especially in a communal breeding bird as the monk parakeet. Sharing a common place, like the colonial nest in this parakeet, did not have an effect on the estimation of heritability of morphological traits. Nest effects were negligible in the univarite animal models since those that included the nest as a random effect presented higher DIC values than models that did not include this effect.

Contrary, we found evidence of maternal effects on morphological traits, being highest in the traits with lower heritability: wing length, bill length and tail length. Price [21] proposed that probably the maternal effects on structural size traits diminish throughout the development and are no longer detectable when the adult size is reached. Nevertheless, there appears to be mounting evidence for enduring maternal effects on some size traits in Darwin’s finches [18].

Maternal effects can have long-term consequences on individuals at evolutionary and ecological time-scales [38, 39]. Multigenerational studies on house finches [40, 41] have highlighted how phenotypic plasticity in maternal effects can have ecological consequences by enabling the colonization of new environments; morphological traits that evolved most rapidly are those that have the greatest maternal and environmental effects [41]. Although we did not evaluate the phenotypic change across generations, we suggest that maternal effects may play a key role on the magnitude and trajectory of the morphological traits in monk parakeets.

### Phenotypic and genetic correlations

Positive phenotypic correlations were high for wing length-tail length (r = 0.71) and wing length-bill length (r = 0.71), and from moderate to low but significant in most of the remaining traits. Those high correlations would indicate strong evolutionary constraints among morphological traits, particularly with those related to flight requirements. It is important to highlight that phenotypic correlations are not always good indicators of genetic correlations because *Vpe* (permanent environment variance), which is a very special case of common environment effect, and maternal effects may obscure such a correlation [42].

However, we also found high genetic correlations between two groups of traits whose phenotypic correlations were from moderate to high, and significant: a) among wing length and bill length, bill width and tail length and b) among the last three traits together. Of the eight comparisons with significant phenotypic correlation in our sample, six presented positive and significant genetic correlations, implying that the observed correlations are due, in part, to additive genetic variance.

Our results are in line with Teplisky et al. [9], who found that genetic correlations among four traits (weight, wing length, tarsus length and bill length) in multigenerational data sets of seven bird species averaged 0.35 (ranging from 0 to 0.76), indicating that genetic correlations can impose significant constraints on the evolution of avian morphology.

The morphological traits assessed in monk parakeet are, in general, to be moderately constrained in terms of evolutionary potential due to the significant values of genetic correlations, except for weight and wing length, and weight and bill width.

### Assortative mating by morphological traits

Our results also suggest the occurrence of assortative mating for weight and bill length, as heavier males tend to mate with heavier females, and males with larger bills tend to mate with females with larger bills. Assortative mating in body condition was evidenced in the Neotropical burrowing parrot *Cyanoliseus patagonus* [43]. However, as pointed out by these authors, a possible explanation for the observed correlation could be that burrowing parrots form long-lasting pair-bonds from an early age. However, all the traits measured in the monk parakeet (this study) show a significant heritability value and therefore could be the focus of mate choice and sexual selection. Therefore, active mate choice or male-male competition by monk parakeets might not be discarded.

## Conclusions

In conclusion, our results indicate the heritability of the studied traits, and therefore the possibility of being shaped by natural selection in response to changing environmental conditions (including global climate change) in both the native and invasive species range. Further long-term studies would be needed to estimate selection coefficients and disentangle the contribution of phenotypic plasticity and selection on the behavioral and morphological traits involved in response to environmental changes by monk parakeets.

## Supporting information

S1 Table Summary statistics for the entire genetic pedigree. (DOCX)

S2 Table Narrow-sense heritability (*h*^*2*^) and maternal effect (*me^2^*) of six morphological traits in the monk parakeet based on animal models (additive genetic and maternal effect). CI: Confidence interval.
(DOCX)
