# Supplementary material for: Quantitative genetics in the monk parakeet (Myiopsitta monachus) from central Argentina: Estimation of heritability and maternal effects on external morphological traits
Source: PLoS One. 2018 Aug 3;13(8):e0201823. doi: 10.1371/journal.pone.0201823 (PMC6075774; doi:10.1371/journal.pone.0201823)
Supplement: S1 Table — (DOCX) [file pone.0201823.s001.docx]

**S1 Table. Summary statistics for the entire genetic pedigree.**

|  | Counts |
| --- | --- |
| records | 219 |
| maternities | 133 |
| paternities | 133 |
| full sibs | 159 |
| maternal sibs | 224 |
| maternal half sibs | 65 |
| paternal sibs | 159 |
| paternal half sibs | 0 |
| maternal grandmothers | 23 |
| maternal grandfathers | 23 |
| paternal grandmothers | 19 |
| paternal grandfathers | 19 |
| maximum pedigree depth | 2 |
| founders | 86 |
| mean maternal sibship size | 3.325 |
| mean paternal sibship size | 2.333 |
| mean pairwise relatedness | 0.0105 |
