# Supplementary material for: Quantitative genetics in the monk parakeet (Myiopsitta monachus) from central Argentina: Estimation of heritability and maternal effects on external morphological traits
Source: PLoS One. 2018 Aug 3;13(8):e0201823. doi: 10.1371/journal.pone.0201823 (PMC6075774; doi:10.1371/journal.pone.0201823)
Supplement: S2 Table — CI: Confidence interval. (DOCX) [file pone.0201823.s002.docx]

**S2 Table. Narrow-sense heritability (*h^2^*) and maternal effect (*m_e_^2^*) of six morphological traits in the monk parakeet based on animal models (additive genetic and maternal effect). CI: Confidence interval.**

| **Trait** | ***h^2^*** | **95 % CI** | ***m_e_^2^*** | **95 % CI** |
| --- | --- | --- | --- | --- |
| **Weight** | 0.314 | 0.005 to 0.679 | 0.348 | 0.047 to 0.618 |
| **Wing length** | 0.042 | 0.002 to 0.114 | 0.800 | 0.701 to 0.891 |
| **Tarsus length** | 0.122 | 0.004 to 0.390 | 0.245 | 0.010 to 0.440 |
| **Bill length** | 0.058 | 0.003 to 0.155 | 0.77 | 0.659 to 0.869 |
| **Bill width** | 0.127 | 0.003 to 0.426 | 0.322 | 0.071 to 0.552 |
| **Tail length** | 0.023 | 0.002 to 0.060 | 0.899 | 0.843 to 0.946 |
